# Microbial Influence on Immune Checkpoint Inhibitor Therapy in Non-Small Cell Carcinoma: The Gut–Lung-Immune Axis

**DOI:** 10.3390/cancers18121948

**Published:** 2026-06-16

**Authors:** Haroon Ali, Bingqing Xie, Jun Yang, Urooba Nadeem

**Affiliations:** 1 Department of Internal Medicine, University of Texas Southwestern, Dallas, TX 75390, USA; haroon.ali@utsouthwestern.edu (H.A.); bingqing.xie@utsouthwestern.edu (B.X.); jun.yang@utsouthwestern.edu (J.Y.); 2 Department of Pathology, University of Texas Southwestern, Dallas, TX 75390, USA

**Keywords:** gut–lung-immune axis, microbiome, immune-checkpoint inhibitors, non-small cell carcinoma

## Abstract

Lung cancer, particularly non-small cell lung cancer (NSCLC), remains the leading cause of cancer mortality worldwide. While immune checkpoint inhibitors (ICIs) have revolutionized treatment, primary and acquired resistance, and immune-related adverse events (irAEs) limit their therapeutic efficacy. Recent evidence highlights the gut and local microbial communities as a modifiable determinant of NSCLC outcomes, especially in the context of ICI use. Emerging data support the concept of a gut–lung-immune axis, a tridirectional communication pathway, in which gut and lung microbial communities influence local and systemic antitumor immunity through immune cell trafficking, cytokine signaling, and microbial-derived metabolites. In this review, we synthesize current clinical and mechanistic studies examining the role of gut, tumor-resident, and circulating microbiota in shaping ICI efficacy and toxicity in NSCLC. Distinct gut and tumor microbial signatures, such as the abundance of *Akkermansia muciniphila* and *Bifidobacterium,* correlate with improved ICI response, whereas dysbiosis promotes immune suppression, resistance, and irAEs. Additionally, we highlight emerging microbial-based biomarkers, including fecal microbial profiles, circulating microbial DNA, and composite tools such as TOPOSCORE, which show promise for predicting response, toxicity, and optimal treatment duration. Overall, these findings underscore the gut–lung-immune axis as a key regulator of immunotherapy outcomes in NSCLC and suggest that microbiome-informed strategies may enable more precise, effective, and safer personalization of ICI therapy.

## Simple Summary

Non-small cell lung cancer (NSCLC) is the leading cause of cancer-related death worldwide. Although treatments such as immune checkpoint inhibitors (ICIs) have improved outcomes, only a small proportion of patients respond to these drugs, and most eventually develop drug resistance or life-threatening side effects. Recent research shows that microorganisms that reside in our bodies (microbiota) function as a dynamic system that influences immune responses. Certain beneficial microbes are associated with better responses to ICIs, while an imbalance in microbial communities (dysbiosis) is linked with treatment resistance and increased toxicity. The gut and lung microbial communities communicate through a pathway known as the gut–lung-immune axis, which is poorly understood. In this review, we summarize the current literature on this topic. Select microbiota from this axis can be used as biomarkers to predict ICI response and may also be targeted to improve outcomes in specific patient populations.

## 1. Introduction

Lung cancer remains the leading cause of cancer-related mortality worldwide, accounting for over 1.8 million deaths annually [1]. Non-small cell carcinoma (NSCLC) accounts for about 85% of lung cancers and is primarily subdivided into adenocarcinoma and squamous cell carcinoma [2]. Immune checkpoint inhibitors (ICIs), antibodies targeting PD-1/PD-L1 and CTLA-4 inhibitors, have significantly improved NSCLC survival, especially for patients with locally advanced or metastatic disease [3,4,5].

Despite these advances, individual responses to ICIs remain highly heterogeneous, and durable clinical benefit is observed only in a subset of patients [3,4]. Therapeutic efficacy is frequently limited by both primary and acquired drug resistance, as well as immune-related adverse effects (irAEs) resulting from off-target immune activation [5,6]. These limitations have spurred interest in identifying patient-specific and tumor-extrinsic factors that may influence ICI responsiveness and toxicity.

Established predictive biomarkers for ICI such as high-tumor mutational burden, PD-L1 positivity by immunohistochemistry, and KRAS/p53 status are limited in their predictive power and constrained by limited tissue availability [7,8]. Of the host-related variables, ECOG performance status, age, and prior treatments and systemic immunity are increasingly recognized as critical determinants of immunotherapy outcomes [7,8,9]. Among these variables, the host microbiome has emerged as a particularly compelling factor because of its profound immunomodulatory capacity and its potential for therapeutic manipulation [10,11,12,13]. The microbiome encompasses not only the commensal microorganisms residing on mucosal and epithelial surfaces but also their collective genomes and metabolic products [14,15].

The gastrointestinal tract harbors the most diverse and complex microbial ecosystem in the human body, which plays a central role in regulating both local and systemic immune responses [11,13]. In addition to the gut, distinct microbial communities have co-evolved as resident microbiota in other organs [15,16]. Moreover, microbial-derived metabolites and immune signaling pathways originating in the gut can influence distant organs, including the lungs [16,17].

**Figure 1:**
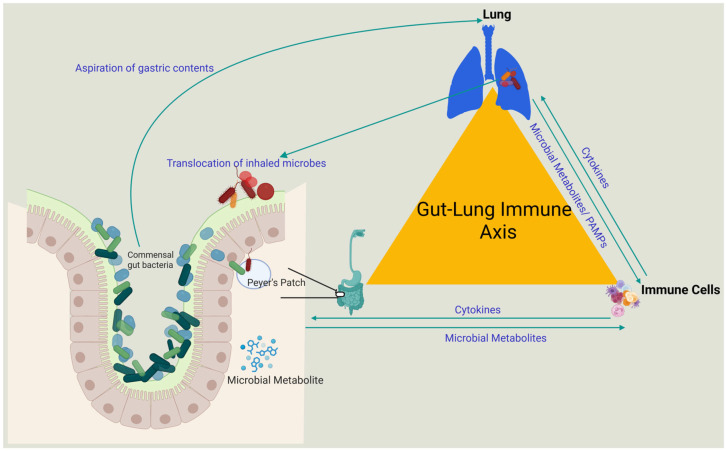
**Conceptual overview of the gut–lung-immune axis.** Schematic illustrating the tridirectional communication between the gut and lungs, mediated by the microbiome and immune system. **Microbial translocation (inhalation and aspiration)** directly links the gut and lung compartments. **Systemic signaling** is maintained through the systemic circulation of **cytokines** and **microbial metabolites**. The “gut–lung-immune axis” (center triangle) represents the integrated feedback loop of gut microbes influence systemic immune responses to regulate immune homeostasis within the lung microenvironment. Created in BioRender. Nadeem, U. (2026) https://BioRender.com/wrqq01a (accessed on 24 May 2026).

Although once considered sterile, the lung is now recognized to harbor distinct, low-biomass resident microbial communities that contribute to immune homeostasis [16,17]. Emerging data highlight crosstalk between the local lung and gut microbial communities, mediated primarily through immune cell trafficking, cytokine signaling, and circulating microbial metabolites [11,18,19]. This tridirectional communication axis, the “gut–lung- immune axis” appears to play a critical role in maintaining pulmonary immune homoeostasis under physiological conditions and in shaping immune responses during malignancy [20] (Figure 1). Numerous studies suggest that host microbiome profiles can impact ICI efficacy, predict response, influence irAEs, and potentially be manipulated to improve outcomes [21,22,23].

Here, we discuss the current evidence defining the role of gut and tumor microbiota in NSCLC treated by ICIs. We also discuss the key microbial taxa associated with the therapeutic response and mechanisms by which they influence efficacy and toxicity. Finally, we consider the current challenges and opportunities associated with these microbiota studies.

## 2. Gut Microbiota and NSCLC

The gut microbiota refers to the trillions of microbes (bacteria, fungi, viruses, archaea, etc.) that reside within the gastrointestinal tract and contribute to host metabolic, inflammatory, and immune homeostasis [15,24]. Disruption of this microbial ecosystem, termed dysbiosis, may arise from environmental factors such as diet, medications, smoking, or lifestyle choices and has been increasingly implicated in carcinogenesis and cancer progression [25,26].

Emerging evidence links gut microbiota composition with the development, progression, and prognosis of non-small cell lung cancer (NSCLC) [27,28,29,30,31,32]. Compared with healthy individuals, patients with NSCLC exhibit altered fecal microbial profiles characterized by reduced abundance of *Firmicutes* and *Proteobacteria* and relative enrichment of *Bacteroidetes* and *Fusobacteria*. Liu et al. demonstrate that the altered *Firmicutes*/*Bacteroidetes* ratio is associated with circulating tumor burden markers such as carcinoembryonic antigen (CEA) in NSCLC patients [29]. Subsequent studies by Zhuang et al. reported increased levels of *Enterococcus* and decreased *Bifidobacterium* in lung cancer patients compared to healthy controls [30].

Gut microbial composition also varies according to disease stage and histologic subtype. Early-stage NSCLC is associated with enrichment of *Lactobacillus*, whereas advanced disease is characterized by increased abundance of *Escherichia coli* and *Bacillus*. Composition also varies by histology; lung adenocarcinoma is enriched in *Fusicatenibacter saccharivorans* and *Roseburia*, whereas squamous cell carcinoma is associated with increased *Proteobacteria*, *Bacteroides*, and *Enterobacteriaceae* [31]. Furthermore, Zheng et al. showed that 13 taxa differentially expressed in the stool could diagnose lung cancer with 97.6% accuracy, thus suggesting that gut microbiota can serve as a robust biomarker [32].

### Gut Microbiota, ICI and Gut–Lung-Immune Axis

Substantial clinical evidence indicates that gut microbiota composition influences the efficacy of ICIs in NSCLC, which is summarized in Table 1. Epidemiological studies demonstrate that broad spectrum antibiotics, which markedly diminish gut microbiota diversity, are associated with poorer responses to ICI efficacy and reduced overall survival [33,34]. This is attributed to the impaired immune homeostasis, leading to a “colder” tumor immune milieu with reduced cytotoxic T-cell infiltration [35,36].

Preclinical models support a potential causal role for gut microbiota in modulating ICI responsiveness. Fecal microbiota transplantation (FMT) from ICI-responders [21,49,58,59] into non-responders reduces tumor growth in germ-free and antibiotic-treated animals. In animal models, *Bacteroides* are enriched in responders, whereas increased *Ruminococcus* is associated with non-responders [59]. In the same animal model, recolonization with *Akkermansia muciniphila* (alone or with *Enterococcus hirae*) can overcome anti-PD-1 resistance, through IL-12-dependent activation of CCR9^+^ CD4^+^ T cells [21]. Clinical cohorts mirror these findings; ICI responders tend to have greater microbial diversity [38,60], accompanied by increased circulating memory CD8^+^ T cells and enhanced natural killer cell activity [37,43,61]. Taxa enriched in responders include *Akkermansia*, *Lactobacillus*, *Blautia*, *Faecalibacterium* and *Ruminococcaceae*, many of which are known to promote dendritic cell activation and T-cell trafficking into tumors [21,43,62]. Conversely, genera such as *Sutterella* and *Bilophila* have been linked to ICI resistance through induction of chronic inflammation and immune exhaustion [43].

Nonetheless, the clinical evidence remains fragmented, with contradictory findings regarding the tumor-promoting versus tumor-suppressive roles of specific microbes [21,62]. For instance, increased abundance of *Akkermansia municiphila* correlates with improved ICIs-response; its levels follow a “Goldilocks” principle. If increased above 4.8%, they are associated with the shortest survival, “normal” abundance (below 4.8%) exhibits the longest median survival, and the complete absence of the species lies between these extremes. This dichotomy underscores that more of a “good” microbe is not always better, and that microbes function collectively as a community to maintain host homeostasis [48,63]. Similarly, high baseline *Bifidobacterium breve* abundance is associated with a longer progression-free survival (PFS) in Asian cohorts receiving anti-PD-1 plus chemotherapy [64], but a European cohort found no survival benefit after adjusting for confounders [65]. These discrepancies may reflect strain-level differences that current metagenomic sequencing cannot distinguish between. Subspecies or strain-level differences can decisively alter immunomodulatory effects, and even closely related strains can exert contrasting effects [58]. For example, two *Bifidobacterium* strains (K57 and K18) showed beneficial synergistic effects with anti–PD-1 therapy, whereas other strains did not. The high genomic similarity between strains (~99%) and limitations in bioinformatics pipelines further complicates translation to clinical practice [58,66]. These findings highlight the limitations of current sequencing approaches and the need for higher-resolution functional analyses.

Currently, only three biomarkers (tumor mutation burden, microsatellite instability, and deficient DNA mismatch repair) are FDA-approved to predict response to ICIs, but their performance is limited [39,67,68]. Small biopsies in lung cancer patients often lead to tissue exhaustion during routine diagnostics, before either of these biomarkers can be obtained. Given these limitations, fecal microbial signatures have emerged as a promising noninvasive alternative [41,42,44,47,50,52,53,54,56,57,69,70]. Specifically, fecal *Akkermansia* levels have shown promise as a stronger predictor of overall survival than PD-L1 expression [48,70]. Derosa et al. developed TOPOSCORE, a novel metric designed to predict ICI response and resistance. They integrated two primary components: a ratio of species-interacting groups (SIGs), 37 bacteria associated with resistance, and 45 bacteria associated with response, and the relative abundance of *Akkermansia muciniphila* [71]. In addition to response, TOPOSCORE can also determine the optimal duration of ICI therapy. Although most clinical trials set ICI duration for advanced NSCLC as 2 years, criteria for safely discontinuing ICIs remain undefined [35,51]. Across the analyzed biomarkers, including radiology, gut microbiota shows the strongest association with PFS in 24 months. For patients reaching this milestone without disease progression, TOPOSCORE could serve as a clinical tool to identify those who can safely discontinue therapy without sacrificing long-term benefits [51].

Beyond taxonomic composition, gut microbial-derived metabolites also influence patient-specific ICI responses by modulating local and systemic immune responses through the gut–lung-immune axis [45,46,50,55,72]. Higher levels of SCFAs, butyrate, propionate, and acetate are associated with favorable ICI responses and longer PFS. SCFAs, especially butyric acid, enhance antitumor immunity by promoting CD8^+^ T-cell activity by producing antitumor cytokines (IL-17, IFN-γ, and IL-10), and upregulate the expression of PD-1 and CD28 on these T cells. They also activate dendritic cells and promote cancer cell apoptosis. These findings align with data demonstrating that SCFA-producing bacteria, such as *F. prausnitzii* and *Ruminococcaceae*, are increased in NSCLC ICI responders [44,47]. Conversely, enrichment of alcohol- and aldehyde-producing bacteria has been observed in non-responders, further underscoring the functional relevance of microbial metabolism [40,45,55,72].

The predictive landscape extends beyond the bacteriome. Eukaryotes, viruses, and archaea also show marked differences between ICI responders and non-responders. For example, enrichment of three eukaryotes (*Nemania serpens*, *Hyphopichia pseudoburtonii*, *Eimeria brunetti*, *Aspergillus tamarii*, *Fusarium anguioides*), and one virus (crAssphage cr127-1) is associated with prolonged PFS. These associations appear independent of age and gender, likely reflecting increased CD8^+^ T-cell activity in the tumor microenvironment, but the biological relevance of this association remains incompletely understood. While individual heterogeneity remains a challenge, these multi-kingdom signatures confirm that the entire microbial community, not just bacteria, participates in the gut–lung-immune axis [73]. Collectively, these findings establish the gut microbiota as a dynamic regulator of immunotherapy efficacy in NSCLC, acting through both immune modulation and metabolite-mediated signaling along the gut–lung axis.

## 3. Lung Resident Microbiome and NSCLC

Traditionally considered sterile, the lung microbiome has only recently been identified using culture-independent sophisticated sequencing techniques [16,74,75]. The lung harbors a low biomass of microbes per gram of tissue (10^3^ to 10^5^) compared to the gut (10^11^ to 10^13^). This low biomass reflects the unique selective pressures of the lung environment, such as oxygen tension, pH, surfactant levels, mucociliary clearance, and the activity of alveolar macrophages. These factors restrict large-scale colonization and favor transient, low-biomass microbial communities. Despite the lower density, the airway microbiome is a meaningful part of the respiratory ecosystem and is intricately linked to the host immune system [16,20,75].

In a healthy, eubiotic state, the lower respiratory tract is dominated by four major bacterial phyla: *Bacteroidetes*, *Firmicutes*, *Proteobacteria*, and *Actinobacteria*. Dickson et al. proposed that microbiome homeostasis is maintained through three dynamic processes: migration (primarily from the oropharynx and gut), elimination (via mucociliary clearance and immune surveillance) and reproduction. In the eubiotic state, community composition is largely shaped by the balance of migration and elimination. However, in dysbiosis or disease state, altered regional growth conditions support the rapid reproduction of specific taxa, potentially driving immune dysregulation and oncogenesis [16,20].

Emerging evidence implicates lung-resident and intratumoral microbiota in the initiation and progression of lung cancer through immune modulation [76,77,78,79,80,81,82,83,84]. Fluorescence in situ hybridization and sequencing studies have confirmed the presence of bacteria within tumor cells and the surrounding stroma, demonstrating that these microbial signals are not artifacts of contamination [78]. Although the origin of the intratumoral microbiota in the lung remains contentious; three main mechanisms are gaining credence: (1) invasion through a disrupted respiratory mucosal barrier, particularly in smokers or patients with chronic inflammation; (2) migration from adjacent lung parenchyma; and (3) hematogenous dissemination of gut-derived microbes or metabolites facilitated by the characteristically leaky tumor vasculature [79,80]. Once established, the immunosuppressive and hypoxic tumor microenvironment supports a persistent microbial colonization [80].

Distinct, specialized roles of microbes are now being recognized depending on whether they reside in the tumor cells or in the extracellular environment. Intracellular microbes can directly influence cell division and mitotic activity, while evading immune surveillance [85,86,87]. Conversely, extracellular microbiota modulates immune responses and metastatic potential by interacting with endothelial and immune cells, thereby influencing angiogenesis, immune infiltration, and metastatic potential. Single-cell transcriptomic data have identified bacterial and fungal signals that are distributed across epithelial tumor cells, immune cells, and stromal cells, with tumor cells exhibiting the highest microbial burden. Moreover, these microbes are not mere bystanders; instead, they actively influence the tumor transcriptome and surrounding microenvironment [13,88,89].

Analogous to other malignancies, intratumor bacteria dominate the microbial landscape in NSCLC, while fungi, viruses and protozoa are present in a minority [74,89,90,91]. However, unlike most tumors, where bacterial density within tumor cells exceeds that of adjacent normal cells, NSCLC does not exhibit a significantly elevated bacterial load in cancerous cells, though the tumor-associated communities are less diverse. This reduced diversity suggests selective pressures favoring specific taxa with tumor-promoting or immune-modulating functions [90,92].

Initial studies used oral (saliva/sputum) and bronchoalveolar lavage (BAL) samples as surrogate markers for the intratumor microbiome [93,94]. These studies consistently report reduced alpha diversity in NSCLC patients and identified oral microbial signatures capable of distinguishing histologic subtypes, predicting disease stage, and correlating with specific oncogenic alterations [95]. Genera such as *Capnocytophaga* and *Veillonella* differentiate squamous cell carcinoma and lung adenocarcinoma, with diagnostic accuracy of 0.86 and 0.80, respectively [96]. Significant associations have also been observed oral microbiota and diagnostic tissue immunohistochemical markers: CK7 and TTF-1 correlate with *Enterobacteriaceae*; while Napsin A associates with genus *Blastomonas* [95]. Overgrowth of salivary *Granulicatella* and *Actinobacillus* correlated with early stage, while *Actinomyces* are increased in advanced NSCLC [97,98,99]. Notably, detection of salivary *Pseudomonas aeruginosa* correlates with brain metastases in NSCLC patients [100], and enrichment of salivary *Parvimonas* is associated with lymph node metastasis and EGFR mutations [99].

**Figure 2:**
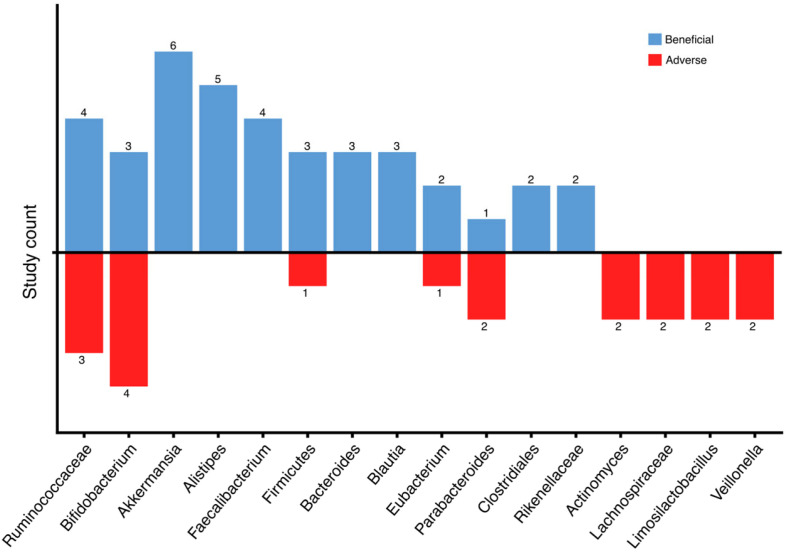

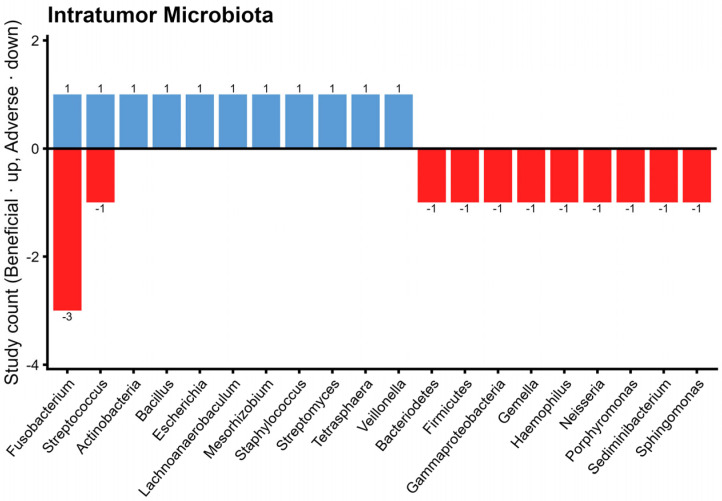
Comparing the effect of similar organisms on ICI efficacy in different studies.

Collectively, these studies illustrate the significant a possible causal role of oral microbiota in lung cancer oncogenesis and progression [18]. Mechanistically, *Veillonella* activates the inflammasome through NLRP1, IL-1β, IL-18, CASP1, while *Streptococcus* promotes CD8^+^ T-cells and Th17 cells. Together, these microbes synergistically activate the PIK3-ERK pathway, a key driver of lung carcinoma proliferation, highlighting potential relevance to PI3K-targeted therapies. However, specific taxa may exert paradoxical physiologic effects [101]. For instance, *Rothia* is associated with a favorable immune microenvironment characterized by increased infiltration of CD4^+^ and CD8^+^ T cells yet is paradoxically linked to decreased survival and increased metastatic potential in squamous cell carcinoma [100]. We compare the gut and intratumor microbiota effects on ICI efficacy in Figure 2.

While oral samples and BAL are easily accessible, direct quantification of intratumor microbes can provide a precise view of the microbes interacting with the tumor and its tumor microenvironment [74]. Unsurprisingly, studies from lung tissue yield a lower percentage of the airway bacteria, and the microbial signatures between the tissue and other sample types are considerably different, reflecting site-specific ecological pressures [102,103,104,105,106,107,108]. Nonetheless, similar to the oral and gut microbiota, associations between epidemiological and clinical features in the lung tissue microbiota have emerged, but these relationships are often more nuanced.

Large-scale studies indicate that early-stage NSCLC does not exhibit a consistent microbial signature, particularly among non-smokers [104]. In contrast, advanced disease is characterized by distinct microbial patterns associated with prognosis and survival. *Thermus* is enriched in advanced-stage tumors, while increased abundance of *Actinomycetales* and *Pseudomonadales* correlates with reduced disease-free survival in stage II NSCLC [109]. *Veillonella*, *Tetrasphaera*, and *Megasphaera* have been proposed as microbial biomarkers for lung cancer detection, and elevated levels of *Roseburia*, *Veillonella*, *Prevotella*, *Streptococcus*, and *Blautia* in tumor tissue are associated with poor outcomes [3]. A multibacterial signature comprising *Haemophilus parainfluenzae*, *Serratia marcescens*, *Acinetobacter jungii*, and *Streptococcus constellation* predicts 2-year survival with high accuracy. Additional studies report increased *Proteus* and *Bacteroides*, accompanied by reduced *Renibacterium*, in patients with lymph node metastasis [74,110].

Intratumoral microbial composition also varies by histologic subtype. Microbial α-diversity is higher in squamous cell carcinoma than in adenocarcinoma [102,111]. *Lactobacillus*, *Leptospira*, and *Mesorhizobium* are enriched in squamous tumors, whereas *Neisseria*, *Mycobacterium*, and *Bacteroides* predominate in adenocarcinoma. Smoking status further shapes these communities; *Acidovorax* is enriched in smokers and is particularly prevalent in TP53-mutant squamous cell carcinoma [106].

Notably, genera *Prevotella* and *Veillonella* are detected concurrently across oral, lung and gut samples [93,94]. However, several taxa exert opposing effects depending on anatomical location. For instance, *Bacteroidetes* species (excluding *Prevotella*), *Spirochetes*, and *Synergistetes* are associated with reduced NSCLC risk when present in saliva but predict poor prognosis when enriched intratumorally. Similarly, *Fusobacteria ssp.* are decreased in the oral microbiome of NSCLC patients yet when increased within tumors, they correlate with adverse outcomes. Butyrate-producing bacteria such as *Roseburia* further illustrate this context-based effect of microbes. Enrichment of these taxa in the gut microbiota is associated with immunoprotective effects, including increased CD8^+^ T-cell infiltration. In contrast, when enriched within the tumor, these microbes and intratumoral butyrate can promote tumor invasion by upregulating *H19* and *MMP15*, inducing M2 macrophage polarization, and increasing interferon-γ production by CD4^+^ and CD8^+^ T-cells. These processes ultimately contribute to effector T-cell depletion and tumor progression [18,112].

In addition to bacteria, lung tumors, especially squamous cell carcinoma, harbor a significantly higher fungal burden and diversity [113,114,115]. Fungi are typically localized within the tumor-associated macrophages. At the genus level, *Blastomyces* and *Talaromyces* are enriched in lung cancer groups, whereas at the species level *Aspergillus sydowii* and *Talaromyces marneffei* correlate with NSCLC. Smoking is strongly linked to increased intratumoral fungal diversity and enrichment of *Aspergillus* and *Agaricomycetes*, with additional variation observed across normal lung tissue, primary tumors, and metastatic lesions [116].

On the other hand, the data regarding viral involvement in NSCLC is sparse [117,118]. Current evidence suggests that lung cancers neither harbor distinct viral organisms nor are they different from normal adjacent tissue [119]. Analysis from RNA sequencing from TCGA reveals that *pegivirus*, *anellovirus*, *human endogenous* retrovirus, and polyomavirus were detected in NSCLC patients. The Epstein–Barr virus has been identified in a subset of pulmonary lymphoepithelioma like carcinoma, a rare NSCLC subtype. Human papillomavirus type 16 has also been detected in lung cancer tissues; however, no association with survival or tumor immune features has been demonstrated [120].

### Intratumor Microbiota, ICI, and Gut–Lung-Immune Axis

We reviewed the available literature in intratumoral microbiota in lung cancer tissue and summarized the most altered species associated with ICIs across different sample types [Table 2]. While the gut microbiome–cancer connection is relatively well characterized, the functional contribution of tumor-associated microbiota to ICI treatment outcomes is still poorly understood [119,121,122,123,124,125,126].

Several studies describe that lung microbial diversity decreases following ICI treatment, characterized by reductions in *Actinomyces*, *Bacteroidetes*, *Bifidobacterium*, and *Prevotella*. Notably, this decline in diversity is often more pronounced among patients who derive the greatest clinical benefit from ICIs [123,124], suggesting selective immune-mediated pressures on intratumoral microbial communities [124,125].

Specific intratumoral taxa have emerged as potential biomarkers for ICI response. Jang et al. reported that *Veillonella dispar* was dominant in ICI responders [119], while shotgun metagenomic analysis has identified *Tetrasphaera* and *Mesorhizobium* as being strongly associated with favorable immune responses [124]. Tumors characterized by high *Fusobacterium* abundance exhibit reduced expression of cytotoxicity-related genes, IFN-γ signaling, and MHC class II molecules, consistent with an immunosuppressive tumor microenvironment [125,126].

ICI success is dependent on pre-existing adaptive tumor immunity and infiltration of functional, cytotoxic T cells, both of which are modulated by local microbiota [126]. Taxa such as *Firmicutes*, *Actinobacteria*, *Moraxella*, *Provetella*, and *Veillonella dispar* can promote PD-L1 expression and recruit Th17 cells, creating a pro-inflammatory environment [100,121,123]. Although these microbes increase the visibility of the tumor cells to the immune system, their impact on ICI success is complex and context-dependent. For instance, in the short-term, *Provetella* and *Veillonella* skew T-cell differentiation toward the Th17 phenotype and IL-17 production, which alters angiogenesis, helps tumor cells evade apoptosis, and suppresses the cytotoxic CD8^+^ T-cell response [128,131]. Eventually, chronic activation of these pathways in the lung creates a “smoldering” inflammatory state that promotes the epithelial-to-mesenchymal transition, allowing tumor cells to become more invasive and metastasize [132].

On the other hand, taxa such as *Haemophilus influenzae* and *Neisseria perflava* are associated with low PD-L1 expression and can potentially identify tumors less likely to respond to standard immune checkpoint blockade regimens [121]. Other pathogenic bacteria also recruit immune cells and alter immune cell infiltration [130,133]. For instance, *Escherichia*-positive NSCLC exhibits gene expression signatures indicative of enhanced immune cell infiltration [128]. These tumors show higher expression of Granzyme B and key chemokines (CCL20, CXCR2P1, CXCL13, and IL12RB2) associated with cytotoxic T-cell activation and infiltration, suggestive of a pro-inflammatory tumor microenvironment. Conversely, the *Escherichia*-negative tumor samples have higher expression of genes with immune-suppressive functions, such as FREM2, PRKCZ, and USP44 [133].

Additional studies reveal that *Bradyrhizobium* and *Prevotella* are significantly associated with increased CD8^+^ T cells, natural killer cells, and activated dendritic, and additionally are strongly linked with good prognosis [129]. Integration of *Prevotella* with routine clinical blood indicators holds promise as a potential predictive tool in the clinic. Moreover, microbiome-derived metabolites further reshape the immune microenvironment by releasing inflammatory cytokines and chemokines, reducing CD8^+^ T-cell response, and promoting M1 phenotypes macrophages within malignant lesions [131,134,135]. Consistent with findings in the gut, intratumoral *Akkermansia* has also emerged as a key modulator of favorable ICI responses in NSCLC. Immunogenic strains such as *Akkp2261* can restore sensitivity to PD-1 inhibitors by inducing dendritic cells’ secretion of IL-12, thereby promoting the recruitment of CCR9^+^ CXCR3^+^ CD4^+^ T lymphocytes into the tumor microenvironment. Together, these findings suggest that intratumoral microbiota not only reflect immunotherapy responsiveness but actively shape the local immune landscape, positioning them as potential targets for tailoring ICI-based treatment strategies [135].

## 4. Circulating Microbial Products

The circulating microbial bacterial DNA (cmDNA) in the bloodstream has emerged as a promising, minimally invasive tool for diagnosis, staging, and predicting outcomes in NSCLC patients. Unlike traditional tissue biopsies, which are subject to spatial heterogeneity, sampling bias, and tissue exhaustion, cmDNA offers a systemic snapshot of the host–microbe interaction that can be obtained repeatedly with minimal risk [136,137].

Studies indicate that NSCLC patients exhibit distinct circulating microbial DNA profiles compared to healthy controls. Blood samples from NSCLC patients are enriched in cmDNA from *Selenomonas*, *Streptococcus*, *Veillonella*, *Acinetobacter*, and *Pseudomonas* [136]. In contrast, healthy cohorts show higher relative abundance of organisms such as *Fusarium oxysporum* and *Delftia*. Remarkably, cmDNA appears to outperform intratumor microbiota in detecting early-stage small tumors, with one study reporting a sensitivity of 86.5% for stage I and 87.1% for tumors smaller than 1 cm [137].

In addition to diagnostic ability, cmDNA signatures also correlate with immune features within the tumor microenvironment and clinical outcomes. Joint analysis integrating intratumor microbiota and the circulating microbes reveals that a high cmDNA abundance is associated with intratumoral CD4^+^ T-helper cell activity, while low cmDNA burden correlates with greater intratumoral B cell infiltration and improved prognosis [137,138,139].

Moreover, other than microbial DNA, circulating microbial-derived metabolites function as systemic signaling molecules that modulate antitumor immunity along the gut–lung-immune axis [140]. Distinct metabolomic profiles can distinguish between ICI responders and non-responders with high accuracy [141]. Hatae et al. found that combining the levels of circulating microbial metabolites, such as hippuric acid, with mitochondrial activity of CD8^+^ T-cell can accurately identify patients who are likely to benefit from ICI therapy [142]. In another study by Masuhiro et al., patients’ responders to ICIs exhibited significantly elevated CXCL9 levels in both bronchoalveolar lavage and blood, accompanied by greater lung microbiome diversity and increased frequencies of circulating CD56^+^ T-cell subsets [129].

Importantly, several studies suggest that circulating microbial and metabolic markers outperform traditional tissue-based biomarkers, including PD-L1 expression, in predicting ICI response. This observation underscores the value of circulating analytes as dynamic biomarkers that capture systemic immune–microbial interactions rather than static tumor feature alone. Overall, these findings support the integration of circulating microbial DNA and metabolite profiling into a multi-omics framework for patient stratification and therapeutic monitoring. When combined with clinical parameters and immune profiling, circulating microbial products holds substantial promise for refining risk assessment, predicting immunotherapy response, and enabling real-time monitoring of treatment efficacy in NSCLC [81].

## 5. Gut and Tumor Microbiota Influencing irAEs

Although ICIs can dramatically improve clinical outcomes in NSCLC patients, these drugs can pose serious toxicity risks. The most common and dangerous of these are immune-related adverse events (irAEs), triggered by an ICI-induced “inappropriate” immune system activation against the hosts’ own cells [143,144,145]. Paradoxically, when promptly treated, irAEs are linked with longer overall and progression-free survival in NSCLC [146]. Gut and tumor microbiota have emerged as key players in predicting and potentially mitigating these toxicities [142].

The composition of the gut microbiota and functional pathways are distinct between patients who develop irAEs and those who do not. NSCLC patients showed an increase in fecal *Lactobacillus* and/or *Bifidobacterium* in non- or low-grade irAE cases [147]. Other taxa frequently elevated in patients with irAEs include *Agathobacter*, *Lactobacillus*, and *Raoultella* [148]. Studies show that viral infections (e.g., HBV, HPV) within the tumor or host can significantly increase the risk of Grade 3+ serious adverse events during ICI monotherapy [149,150].

Different microbial taxa confer beneficial effects by altering immune cells [150]. For instance, Tan et al. demonstrated that *Lactobacillus rhamnosus* decreased immune-related enteritis by modulating Treg cells in animal models [149]. Preclinical studies also suggest that *Bifidobacterium* supplementation can alleviate colitis in mice receiving ICIs [151], via increased expression of IL-10Ra and IL-10 on intestinal Treg cells [152]. Both *Bifidobacterium* and *Lactobacillus* are believed to promote Treg differentiation and function, aiding in the balance of the immune system to counteract the hyper-activation caused by ICIs [151,153,154,155].

Moreover, organ-specific irAE involvement, e.g., colitis, pneumonitis, etc., often correlates with unique microbial signatures. For instance, immune-related diarrhea in NSCLC patients is associated with enrichment of phyla *Firmicutes* with an accompanying decrease in *Bacteroidetes* [155]. Genera associated with development of immune checkpoint-related colitis include *Faecalibacterium prausnitzii*, *Bacteroides fragilis*, and *Lactobacillus reuteri* [149]. On the other hand, checkpoint inhibitor-related pneumonitis occurs with significantly higher incidence in NSCLC patients receiving ICIs, and can be dose-limiting [156,157]. These patients who develop pneumonitis have higher intratumoral microbial α-diversity and enrichment of *Vibrio*, *Halomonas*, *Mangrovibacter*, *Paracoccus*, *Salinivibrio*, along with a decrease in *Lachnospiraceae* and *Akkermansia* [22,158]. This dysbiosis activates CD8^+^ T activation via lauroylcarnitine, which induces IFN-γ and TNF-α secretion, augmenting drug-related injury [159]. One study describes a microbial signature with increased *Candida* and *Treponema* in BAL to predict and identify checkpoint inhibitor pneumonitis [160].

Gut microbial metabolites can also assist in reducing the incidence of irAEs. SCFAs (acetic acid, propionic acid, and butyric acid) are generally lower in patients with severe irAEs [161,162]. Diets enriched in fiber and omega-3 support SCFA-producing microbes, such as *Bifidobacterium*, and can attenuate the production of pro-inflammatory cytokines and thereby decrease risk [152]. SCFA-rich diets can significantly reduce gastrointestinal toxicity induced by immunotherapy [162,163].

Given that the incidence of irAEs of lung patients treated with checkpoint inhibitor nivolumab may reach up to 51%, targeting the microbiome in combination with ICIs may provide beneficial treatment outcomes [164]. Further prospective, mechanistic studies are needed to translate these findings into routine clinical practice and to clarify how gut and intratumoral microbes interact with host immunity to influence irAEs and treatment outcomes.

## 6. Mechanisms Through Which the Gut-Lung-ImmuneAxis Influences ICIs

**Figure 3:**
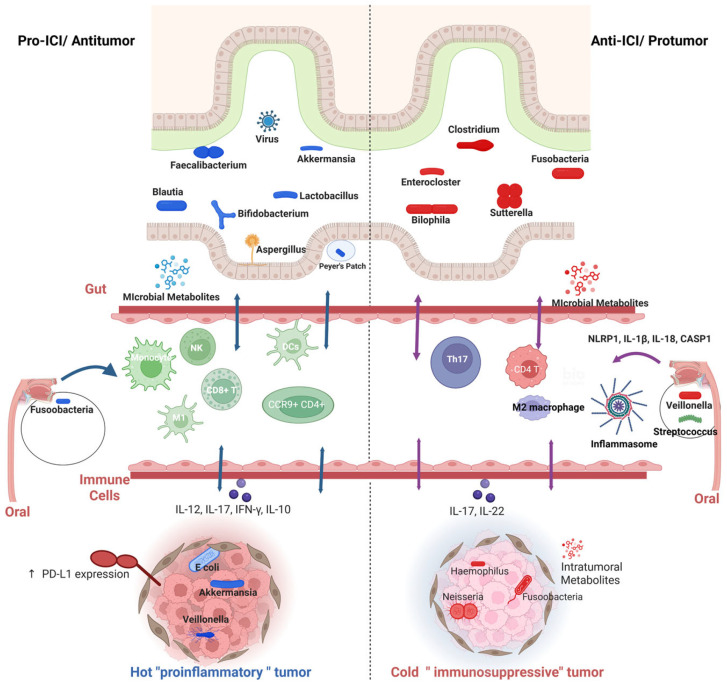
**The gut–lung-immune axis and its modulation of immune checkpoint inhibitor (ICI) efficacy.** Schematic representation of the role of microbial communities in shaping the tumor microenvironment (TME) and determining responses to ICI therapy. **Pro-ICI (Left):** Beneficial gut microbes (*Akkermansia*, *Bifidobacterium*, *Faecalibacterium*, and *Lactobacillus*) promote a “hot,” proinflammatory TME by activating dendritic cells, natural killer cells, M1 macrophages, and CD8^+^ T-cells, which enhance ICI response through IL-12, IFN-γ, and IL-10, and increased PD-L1 expression. **Anti-ICI/(Right):** Dysbiosis (*Clostridium*, *Fusobacteria*, *Sutterella*) contributes to a “cold,” immunosuppressive TME by activating Th17 cells, M2 macrophages, and causing resistance through IL-17 and IL-22. **Oral microbial translocation:** Pathogenic oral microbes (*Veillonella*, *Streptococcus*) activate the inflammasome through NLRP1, IL-1, IL-18, and CASP1, which promote resistance to ICIs. This figure was created using Biorender.

Although anatomically distinct, the gut and lung are both mucosal organs that interface with the external environment and share a common embryologic origin [165,166]. Table 3. (A, B and C) summarizes select gut, oral and intratumoral microbiota, their impact on the immune system and ICI responses. An increasingly attractive hypothesis suggests a bidirectional communication network between these sites, mediated in part by their resident microbiota. Direct communication can occur via translocation of inhaled microbes from the lung into the gastrointestinal tract and the microaspiration of gastric contents into the lungs [165,166,167,168]. Indirect communication involves immune-mediated pathways, involving cytokine signaling and immune cells through mucosal lymphatics and systemic circulation. Together, these processes form a tridirectional, rather than bidirectional, network, the “gut–lung-immune axis.” This axis regulates antitumor immunity and influences both the efficacy and toxicity of ICIs [11,169,170,171] (Figure 3).

Gut derived antigens captured in the gut-associated lymphoid tissue, such as Peyer’s patches, influence innate immunity, and drive B-and T-cell differentiation. Activated immune cells travel to the mesenteric lymph nodes and then to the lung, shaping pulmonary immunity by priming local T cells and promoting regulatory cytokine production [172,173,174,175,176]. For instance, *Hominenteromicrobium* promotes maturation of CD103^+^CD11b^−^ dendritic cells in the gut, which then migrate to the tumor microenvironment, and activate tumor-specific CD8^+^ T cells, promoting PD-L1 expression, and enhancing ICI sensitivity [177,178]. Similarly, *A. municiphila* in the gut correlates with improved ICI response in NSCLC by recruiting CCR9^+^ CXCR3^+^ CD4^+^ T cells.

Local lung microbiota also play a direct role in shaping antitumor immunity. Higher lung microbiota α-diversity correlates with increased CXCL9 secretion and greater CD8^+^ T-cell infiltration within the tumor microenvironment, thereby suppressing tumor growth [179]. Other commensals, such as *Bacteroidales*, are associated with higher circulating regulatory T-cells, myeloid-derived suppressor cells, and blunted cytokine response to ICIs, resulting in a better PFS [180,181]. In the gut, enrichment of *Ruminococcus* is linked to elevated circulating CD4^+^ and CD8^+^ T-cells and enhanced CD8^+^ T-cell tumor infiltration. Within the tumor microenvironment, a higher density of CD8^+^ T-cells is observed in responders versus non-responders, and this infiltration positively correlates with the *Clostridiales*, the *Ruminococcaceae*, and *Faecalibacterium*, while it negatively correlates with *Bacteroidales* [182,183].

Conversely, dysbiosis can disrupt these pathways, leading to poor ICI responses. For example, enrichment of *Enterocloster* spp. disrupts mucosal addressin molecules (e.g., MAdCAM-1) and causes aberrant migration of α4β7^+^ Th17/Treg17 cells to tumor tissues. This leads to significant pulmonary immunosuppression, including reductions in γδ T cells, natural killer cells, macrophages, dendritic cells, monocytes, and neutrophils, in the tumor, thus compromising the efficacy of ICIs and accelerating tumor growth [184,185].

Immune modulation by microbial metabolites is another mechanism [186,187]. Short chain fatty acids (SCFAs), such as butyrate, acetate, and propionate, produced in the gut influence systemic and lung immunity. Butyrate enhances CD8^+^ T-cell-mediated responses via IL-12 signaling and promotes long-term T-cell persistence by reprogramming cellular metabolism toward fatty acid oxidation and glutaminolysis [188]. SCFAs can result in increased tumor immunogenicity by upregulating MHC-1 expression and downregulating inhibitory ligands like CD155, making the tumor more visible to the immune system [108]. While exogenous butyrate often suppresses tumor growth, endogenous butyrate produced by the tumor may occasionally promote metastasis or drug resistance under specific therapeutic conditions. Unlike butyrate and propionate, acetate can have pro-tumorigenic effects, promoting cell survival and immune evasion by upregulating PD-L1 through c-Myc stabilization [108].

Beyond metabolic signaling, gut microbiota may influence ICI efficacy through molecular mimicry [152]. Although central tolerance eliminates most self-reactive T cells, a subset escapes deletion and can be activated by microbial antigens that resemble host or tumor antigens [34]. This cross-reactivity can potentiate antitumor immune responses by enhancing T-cell–mediated cytotoxicity against malignant cells, thereby augmenting responsiveness to checkpoint blockade [148].

In summary, the magnitude of microbiome-mediated effects on ICI are shaped by host genetics, tumor histology, microbial composition, disease stage, and prior antibiotic exposure. Overall, microbiota influence ICI responses in NSCLC through an integrated network: (1) systemic immune priming via gut-derived signals; (2) local tumor microenvironment regulation via gut and intratumoral microbiota; (3) metabolites signaling; and (4) molecular mimicry. Understanding and therapeutically leveraging these pathways may transform the microbiome from a prognostic indicator into a clinically actionable target for optimizing immunotherapy in NSCLC.

## 7. Challenges and Future Opportunities

Although the evidence discussed above demonstrates a clear role of the microbiome in NSCLC, particularly in the context of ICI therapy, the findings across studies and sample types remain inconsistent and, in many cases, poorly reproducible. Exploring these discrepancies is critical for translating these findings into clinically actionable strategies. The major challenges and their proposed solutions are summarized in Table 4.

A significant hurdle is that most mechanistic insights are derived from animal models, rather than validated clinical studies. Estimates suggest that only 2–4% of microbial taxa are shared between mice and humans, underscoring the limited translatability of preclinical findings [189,190]. Even among clinical studies, baseline microbial composition varies significantly across individuals due to host-related variables. For example, distinct microbial diversities are found across racial and ethnic groups, such as variations in the abundance of *Bacteroides* versus *Prevotella* between African American, Caucasian, and Asian cohorts, yet these differences are often underrepresented or inadequately controlled in study designs [165,166,167,168]. Additionally, the microbiome is highly dynamic; certain taxa play distinct roles depending on the tumor stage or the specific anatomical site [169]. Since smaller studies cannot adequately account for these variables, large-scale, multicenter longitudinal analyses are required to identify population-specific microbial signatures.

In addition to biologic confounders, technical biases introduce significant noise that impedes reproducibility and generalizability. Variations in sequencing methods, such as differences in 16S rRNA gene regions targeted, DNA extraction protocols, and sequencing platforms, can result in substantial discrepancies in reported microbial compositions [170,191,192]. Moreover, relying on stool or oral samples as proxies for the gut or intratumoral microbiota fails to fully capture the distinct microbial ecosystems within the gastrointestinal tract or the tumor [192].

Tumor microbiome studies, especially in the lungs, are often limited by ultra-low biomass, making them prone to sequencing noise and DNA contamination from kits and reagents [173]. While this problem can somewhat be mitigated by decontamination algorithms (e.g., decontam, microDecon) and inclusion of multiple negative “blank” controls at every step, standardization across laboratories remains lacking [174,175]. These taxa found in the kits or reagents can be manually removed to identify true intratumor microbiota. Additionally, environmental controls and positive mock communities (known microbial compositions) assess sequencing accuracy and potential cross contamination.

At the level of data interpretation, accurate taxonomic classification of microbial sequences relies on robust databases with diverse microbial representation. Most currently available databases, such as NCBI, were primarily designed for research and contain errors or misannotated sequences. While sharing raw data to public repositories (e.g., via the European Nucleotide Archive or NIH Sequence Read Archive) is increasing, the data is often deidentified and the essential patient-specific information (age, ethnicity, drug use) for clinical validation is missing. Greater transparency in analytical pipelines, including host read removal, denoising steps, and database alignment, is essential to enable meaningful cross-study comparisons [176,177,178]. Machine learning and probabilistic modeling are being introduced to distinguish between true intratumor microbiota and contaminants [193].

Likewise, microbiome studies frequently lack statistical rigor. Many studies are underpowered, and compositional sequencing data are frequently treated as independent quantitative measures despite representing relative, rather than absolute, abundances. This misinterpretation can result in false-positive findings. To address this, researchers are moving toward high-dimensional frameworks specialized in the unique properties of microbial sequencing data rather than remodeling the previously developed tools meant for human sequencing data; however, adoption remains inconsistent. In addition, taxonomic signals are often assumed to reflect underlying functional relevance, yet relatively few studies have directly interrogated microbial function via metagenomics, metatranscriptomics, or metabolomics analyses. This gap limits mechanistic interpretation into how microbial communities influence ICI response in NSCLC [177,194].

Despite its promise, microbiota-targeted interventions have not yet been successfully translated into routine clinical practice. First, there are inconsistent findings across studies. For instance, although *Ruminococcae* and *Bifidobacterium* are often highlighted for their metabolic and immunomodulatory benefits, their associations across independent studies remain highly inconsistent Figure 3. This variability is driven by a multitude of factors, including host-specific differences in dietary fiber intake, regional geography, recent antibiotic use and distinct treatment regimens. Consequently, methodological differences in sequencing techniques (16S vs. Metagenomics), cohort composition, and antecedent antibiotic exposure necessitate cautious interpretation of these microbial markers, emphasizing the need for strain-resolved, context-dependent evaluations in future microbiome research.

In addition to biologic, technical and analytic difficulties in the scientific lab, this translational gap is driven by poorly designed clinical trials and a lack of regulatory and practical barriers [195,196]. FMT, for instance, raises safety concerns, such as significant donor effects, where outcomes depend strongly on the specific microbiota composition of the donor, or unpredictable microbial shifts could lead to adverse effects [22]. Safety, regulatory, and long-term risk considerations pose major hurdles for microbiome-based therapies in oncology. Immunocompromised patients face elevated risk of sepsis and transmission of multidrug-resistant organisms, particularly with inadequately screened fecal microbiota drugs [197]. Probiotics can also cause bloodstream infections or immune complications when used outside tightly controlled clinical settings [198]. Regulatory pathways for live or engineered microbiome products are complex and vary by region, and there is ongoing debate to ensure consistency [195,196]. Additionally, long-term consequences of microbiome therapeutics remain poorly understood; most safety data address short-term outcomes, as possible effects on metabolism, immunity, and neurological function may emerge only with extended follow-up. Overcoming these challenges will require interdisciplinary collaboration among oncologists, pathologists and data scientists.

Finally, the rapid advancement of artificial intelligence offers a powerful way to decode the complex relationship between the microbiome, host immunity, and tumor biology. AI-driven analyses of integrated multi-omics data can improve patient stratification, identify predictive microbial signatures, and uncover previously unrecognized mechanisms of ICI resistance or sensitivity. To ensure clinical utility and ethical use of these advances, these approaches must rely on standardized data collection, transparent algorithms, and rigorous validation.

## 8. Conclusions

Emerging evidence indicates the central role of the gut–lung-immune axis in shaping antitumor immunity and modulating the efficacy and toxicity of immune checkpoint inhibitors (ICIs) in non-small cell lung cancer (NSCLC). If therapeutically beneficial microbial populations can reliably enhance ICI responses in humans, these microbiome-based strategies would potentially be cheaper than combining ICIs with other expensive modalities like stereotactic radiation or tumor vaccines. However, translation to bedside treatment requires identifying precise microbial signatures associated with response, as simple measures of diversity are too nonspecific to serve as reliable biomarkers.

Despite substantial progress, several challenges remain in harnessing microbiota-based therapies to optimize ICI efficacy. Future research should aim to prioritize these three goals: (1) identification of robust microbial and metabolite-based biomarkers predictive of ICI response and toxicity; (2) mechanistic elucidation of how microbiota interact with host immunity and the tumor microenvironment; and (3) translation of these insights into safe, standardized, and clinically actionable interventions. Approaches such as rationally designed probiotics, dietary modulation, microbial metabolites, or carefully controlled microbiota transplantation may ultimately augment the efficacy and safety of immunotherapy in NSCLC.

Overall, the novel preclinical and clinical evidence supports a critical role for the gut microbiome in shaping host immunity and therapeutic response, acting through both local tumor microenvironment interactions and systemic immune pathways. This understanding could inform novel, microbiome-guided strategies to improve cancer outcomes.

## Figures and Tables

**Table 1:** Gut microbes altered in NSCLC patients receiving ICIs. R—responders, NR—non-responders, PFS—progression-free survival, HPD—high progression disease.

Author	Analytic Method	Sample Size	Beneficial	Adverse	Reference
Routy	Metagenomics	37 R, 23 NR	*Akkermansia muciniphila*; *Alistipes* spp., and *Eubacterium* spp.	*Bifidobacterium adolescentis*, *B. longum*, and *Parabacteroides distasonis*	[21]
Katayama	16S rRNA sequencing (V1–V2)	6R, 11 NR	*Lactobacillus*; *Clostridium*; *Syntrophococcus*	*Bilophila*; *Alphaproteobacteria*; *Sutterella*; *Parabacteroides*	[37]
Jin	16S rRNA sequencing (V3–V4)	23 R, 14 NR	*Alistipes putredinis*, *Bifidobacterium longum*, and *Prevotella copri R* Higher diversity and stable microbiota	*Ruminococcus*	[38]
Yin	16S rRNA sequencing (V4)	23 R, 19 NR and PFS	*Akkermansiaceae*; *Enterococcaceae*; *Enterobacteriaceae*; *Carnobacteriaceae*; *Clostridiales Family XI bacterial families*		[39]
Vernocchi	Metagenomics and Metabolomics	7R, 4 NR and 8 controls	*Akkermansia muciniphila*; *Rikenellaceae*; *Bacteroides*; *Peptostreptococcaceae*; *Mogibacteriaceae*; *Clostridiaceae*		[40]
Dora	Metagenomics	46 Long PFS, 16 Short PFS	Short-term PFS: *Firmicutes* and *Actinobacteria*; Long-Term-*Alistipes*, *Barnesiella visceriola*	*Streptococcus* species and *Bifidobacterium*	[41]
Haberman	16S rRNA sequencing (V4)	75 NSCLC patients (50 treated with ICIs), 31 controls	*Akkermansia muciniphila*, *Alistipes onderdonki*, *Ruminococcus*	*Clostridium citroniae*	[42]
Shoji	16S rRNA sequencing (V3–V4)	17 R, 11 NR	*Blautia*	*RF32 unclassified*	[43]
Sitthideatphaiboon	Metagenomics	35 non-HPD, 22 HPD	*Firmicutes*; *Ruminococcaceae*	*Intestinimonas*; *Enterobacteriacea*	[44]
Botticelli	Metabolomics	4 early progression, 7 late progression	SCFAs	ketone and alkane	[45]
Song	Metagenomics	635 PFS ≥ 6 months, 28 PFS < 6 months	*Parabacteroides* and *Methanobrevibacter*	*Veillonella*, *Selenomonadales*, and *Negativicutes*	[46]
Hakozaki	16S rRNA sequencing (V3–V4)	270; R vs. NR at 12 months	*Fusicatenibacter*, *Butyricicoccus*, *Blautia*, *Bifidobacterium*, and *Eubacterium ventriosum*	*Ruminococcaceae UBA1819*, *Prevotellaceae NK3B31 group*, *Oscillibacter*, and *Lactobacillus*	[47]
Derosa	Shotgun Metagenomics	338	*Akkermansia*, *Ruminococcacae*, *Alistipes*	*Veillonella parvula*, *Actinomyces* and *genus Clostridium*	[48]
Newsome	16S rRNA sequencing (V1–V3)	18R, 47NR	*Ruminococcus*; *Akkermansia*; *Faecalibacterium*		[49]
Martini	16S rRNA sequencing (V4)	5 long PFS, 9 short PFS	*Agathobacter*, *Blautia*	*Lachnospiraceae*, *Ruminococcus*	[50]
Bonato	Metagenomics	21 patients with >2 years of ICI, 10 patients stopped treatment at 2 years	Sig 2	Sig1	[51]
Komatsu	16S rRNA sequencing (V1–V2)	12 R, 7 NR	*Bifidobacteriaceae*, *Levilactobacillus brevis*	N/A	[52]
Ren	16S rRNA sequencing (V3–V4) and Mass Spectrometry	41 R, 20 NR	*Faecalibacterium*	N/A	[53]
Charalambous	16S rRNA sequencing (V3–V4)	18 treated, 154 controls	*Bacteroidacaeae*	Firmicutes, Lachnospiraceae and Ruminoccocaceae	[54]
Yang	16S rRNA sequencing and metagenomics	53 R, 53 NR	*Fecalibacterium*, *Subdoligranulum*, *Firmicutes*, *Bacteroides*, and *Faecalibacterium*	*Limosilactobacillus*, *Escherichia-Shigella*, and *Bifidobacterium*	[55]
Dora	Metatranscriptomics	16 long PFS, 13 short PFS	*Bacillota*	*Bifidobacterium*, *Collinsella*, *Limosilactobacillus*, and *Eubacterium Actinomycetota*, *Euryarchaeota*, and *Archea*	[56]
Zhang	16S rRNA sequencing (V3–V4)	25R (stool = 8, stool and saliva = 17), 50NR (stool = 8, and saliva = 40)	*Desulfovibrio*, *Actinomycetales*, *Bifidobacterium*, *Odoribacteraceae*, *Anaerostipes*, *Rikenellaceae*, *Faecalibacterium*, and *Alistipes*	*Fusobacterales*, *Fusobacteriia*, *Fusobacterium*, *Fusobacteria*, and *Fusobacteriaceae*	[28]
He	16S rRNA sequencing (V3–V4)	8 SD, 8 PD	*Escherichia*, *Shigella*, *Akkermansia*, and *Olsenella*	*Anaeroglobus*	[57]

**Table 2:** Intratumor/oral microbes altered in ICI treatment.

Author	Analytic Method	Sample Size	Specimen Type	Beneficial	Adverse	Reference
Jang	16S rRNA sequencing (V3–V4)	3R, 8NR	BAL	*Veillonella dispar*	*Haemophilus influenzae* and *Neisseria perflava*	[119]
Chu	16S rRNA sequencing (V3–V4)	19R, 27NR	BAL	*Actinobacteria*	*Fusobacterium*	[121]
Zapata-Garcia	16S rRNA sequencing (V3–V4)	55 (Stage III/IV); 13R, 42 NR	Saliva	*Lachnoanaerobaculum*, *Fusobacterium*	*Firmicutes*, *Bacteriodetes*, *Gemella*, *Streptococcus*, *Porphyromonas*	[127]
Shoji	Metagenomics	18R, 14NR	Tumor	*Tetrasphaera* and *Mesorhizobium*		[123]
Zhang, Y	16S rRNA sequencing (V3–V4) and metabolomics	17R, 11NR	BAL	*Bacillus*	*Sphingomonas*, *Sediminibacterium*	[126]
Zhang, C	16S rRNA sequencing (V3–V4)	25R, 50NR	Sputum and Stool	*Streptococcus*		[122]
Chen	Metagenomics and metabolomics	17R, 11NR	BAL	*Staphylococcus* and *Streptomyces*		[128]
Masuhiro	16S rRNA sequencing (V3–V4)	6R,6NR	BAL and blood	N/A		[129]
Boesch	16S rRNA sequencing (V3–V4)		Tumor		*Gammaproteobacteria*	[124]
Battagalia		63 NSCLC subsets in 4160 specimens	Tumor	N/A	*Fusobacterium*	[125]
Elkrief		Discovery cohort: 958; Validation cohort: 772	Tumor	*Escherichia*		[130]

BAL—bronchoalveolar lavage, R—responders, NR—non-responders.

**Table 3:** (**A**) gut microbiota. (**B**) oral microbiota and immune effects. (**C**) intratumor microbiota and immune effects and its impact on ICI response.

(A)		
Organism/Taxa	Immune Effect/Mechanism	Impact on ICI Response
*Akkermansia* *muciniphila*	Promotes IL-12 production, activates dendritic cells, recruits CD4^+^ T cells	Improved response
*Bifidobacterium*	Enhances T-cell activation and immune regulation	Improved response (context-dependent based on specific strain)
*Faecalibacterium* (*F. prausnitzii*)	Produces SCFAs → enhances CD8^+^ T-cell activity and cytokine production	Improved response, PFS and OS
*Lactobacillus*	Dendritic cell and T-cell migration to the TME and Treg regulation	Early stage disease; improved response; reduced toxicity
*Ruminococcaceae*	Promotes CD8^+^ T-cell infiltration and immune activation	Improved response
*Alistipes* spp.	Supports immune activation and microbial diversity	Improved response
Most SCFA-producing bacteria	Produce butyrate, acetate → enhance antitumor immunity	Improved response
*Sutterella*	Promotes immune exhaustion and chronic inflammation	Resistance
*Bilophila*	Induces inflammatory signaling	Resistance
*Clostridium* spp.	Promotes immunosuppressive tumor microenvironment	Reduced response
(**B**)		
**Organism/Taxa**	**Immune Effect/Mechanism**	**Impact on ICI Response**
*Veillonella*	Activates inflammasome (IL-1β, IL-18), promotes inflammation	Context-dependent (often adverse)
*Streptococcus*	Promotes CD8^+^ T-cells and Th17 responses	Mixed effects
*Prevotella*	Drives Th17 differentiation and IL-17 signaling	Mixed/may promote tumor progression
*Capnocytophaga*	Associated with tumor subtype-specific immune responses	Diagnostic relevance
*Granulicatella*/*Actinobacillus*	Associated with early-stage disease immune responses	Context-dependent
*Pseudomonas aeruginosa*	Associated with metastatic progression and immune dysregulation	Adverse outcomes
(**C**)		
**Organism/Taxa**	**Immune Effect/Mechanism**	**Impact on ICI Response**
*Akkermansia* *muciniphila*	Promotes IL-12 production, activates dendritic cells, recruits CD4^+^ T-cells	Improved response
*Tetrasphaera*	Associated with favorable immune activation	Improved response
*Mesorhizobium*	Linked to enhanced antitumor immune signatures	Improved response
*Escherichia*	Associated with increased cytotoxic T-cell infiltration and chemokine expression	Improved response
*Veillonella dispar*	Associated with ICI responders in some cohorts	Improved response (context-dependent)
*Fusobacterium*	Suppresses IFN-γ signaling and cytotoxic gene expression	Poor response
*Haemophilus* *influenzae*	Associated with low PD-L1 expression	Poor response
*Neisseria perflava*	Reduced immune activation	Poor response
*Gammaproteobacteria*	Associated with immune suppression	Resistance

PFS: progression-free survival; OS: overall survival.

**Table 4:** Challenges associated with translating microbiome ICI studies into clinical practice and proposed solutions.

Category	Specific Challenge	Proposed Solutions
Biological	Limited translatability of preclinical models (2–4% of microbiota overlap in mice and humans)	Emphasize human-based longitudinal studies; validate mechanisms using patient-derived samples and organoid or ex vivo immune models
	Confounding variables such as geographic location, diet, and host genetics	Conduct multicenter, longitudinal studies, establish large-scale consortia and require the public sharing of raw sequencing data with detailed patient metadata
	Inaccessibility of the lower respiratory tract compared to the gut	Increased use of direct tumor tissue profiling when feasible; integrated analysis across gut, lung, blood, and tumor compartments
	Taxonomic focus (who is there) rather than functional focus (what they are doing)	Design multi-omics (metagenomics, metatranscriptomics, and metabolomics) studies and functional assays
	No definition of “dysbiosis”	Use systems biology to elucidate microbial signatures associated with disease and health states
Technical	Lack of standardization in DNA extraction and 16S rRNA gene PCR protocols	Adopt uniform protocols for sample collection, sequencing, and bioinformatics analysis to improve reproducibility and cross-study comparability
	Contamination from DNA extraction kits and laboratory reagents	Implement ultrapure reagents, certified nuclease-free consumables, and dedicated pre-PCR clean rooms
	Ultra-low microbial biomass in lung tissue leads to high background sequencing noise	Use decontamination algorithms (e.g., decontam, microDecon) and include multiple negative “blank” controls at every step
Analytical	Databases with misannotation and missing information	Cross-reference and harmonize multiple database sources
	Compositional sequencing data interpreted as absolute rather than relative measure	Adoption of microbiome-specific statistical frameworks; rigorous correction for confounders; transparent reporting standards
Microbiome targeted therapies	“Donor effects” in fecal microbiota transplantation (FMT) causing inconsistent results	Develop defined microbial consortia or Live Biotherapeutic Products to replace raw FMT
Clinical Trials	Using descriptive microbiome metrics as endpoints	Microbial therapeutics should be linked to long-term functional endpoints, e.g., metabolites Control for key confounders such as diet and medication
Regulatory	No regulation of microbial products	Address both short-term and long-term effects; studies in humans

## Data Availability

No new data was generated in this study.
